# A Novel Perspective of the Kalman Filter from the Rényi Entropy

**DOI:** 10.3390/e22090982

**Published:** 2020-09-03

**Authors:** Yarong Luo, Chi Guo, Shengyong You, Jingnan Liu

**Affiliations:** 1 Global Navigation Satellite System Research Center, Wuhan University, Wuhan 430079, China; yarongluo@whu.edu.cn (Y.L.); shengyongyou@whu.edu.cn (S.Y.); jnliu@whu.edu.cn (J.L.); 2 Artificial Intelligence Institute, Wuhan University, Wuhan 430079, China

**Keywords:** Rényi entropy, discrete Kalman filter, continuous Kalman filter, algebraic Riccati equation, nonlinear differential Riccati equation

## Abstract

Rényi entropy as a generalization of the Shannon entropy allows for different averaging of probabilities of a control parameter α. This paper gives a new perspective of the Kalman filter from the Rényi entropy. Firstly, the Rényi entropy is employed to measure the uncertainty of the multivariate Gaussian probability density function. Then, we calculate the temporal derivative of the Rényi entropy of the Kalman filter’s mean square error matrix, which will be minimized to obtain the Kalman filter’s gain. Moreover, the continuous Kalman filter approaches a steady state when the temporal derivative of the Rényi entropy is equal to zero, which means that the Rényi entropy will keep stable. As the temporal derivative of the Rényi entropy is independent of parameter α and is the same as the temporal derivative of the Shannon entropy, the result is the same as for Shannon entropy. Finally, an example of an experiment of falling body tracking by radar using an unscented Kalman filter (UKF) in noisy conditions and a loosely coupled navigation experiment are performed to demonstrate the effectiveness of the conclusion.

## 1. Introduction

In the late 1940s, Shannon introduced a logarithmic measure of information [1] and a theory that included information entropy (the literature shows that it is related to Boltzmann entropy in statistical mechanics). The more stochastic and unpredictable a variable is, the larger its entropy is. As a measure of information, entropy has been used in various fields, such as information theory, signal processing, information-theoretic learning [2,3], etc. As a generalization of the Shannon entropy, Rényi entropy, named after Alfréd Rényi [4], allows for different averaging of probabilities through a control parameter α, and is usually used to quantify the diversity, uncertainty, or randomness of random variables. Liang [5] presented the evolutionary entropy equations and the uncertainty estimation for Shannon entropy and relative entropy, which is also called Kullback–Leibler divergence [6], within the framework of dynamical systems. However, higher-order Rényi entropy has some better properties than Shannon entropy by setting the control parameter α in most cases.

The Kalman filter [7] and its variants have been widely used in navigation, control, tracking, etc. Many works focus on combining different entropy and entropy-like quantities with the original Kalman filter to improve the performance. When the state space equation is nonlinear, Rényi entropy can be used to measure the nonlinearity [8,9]. Shannon entropy was used to estimate the weight of each particle from the weights of different measurement models for the fusion algorithm in [10]. Quadratic Rényi entropy [11] of innovation has been used as a minimum entropy criterion under a nonlinear and non-Gaussian circumstance [12] in unscented Kalman filter (UKF) [13] and finite mixtures [14]. A generalized density evolution equation [15] and polynomial-based non-linear compensation [16] were used to improve the minimum entropy filtering [17]. Relative entropy has been used to measure the similarity between the probabilistic density functions during the recursive processes of the nonlinear filter [18,19]. As for the nonlinear measurement equation with additive Gaussian noise, relative entropy can be deduced to measure the nonlinearity of the measurement [20], and can also be used to measure the approximation error of the *i*-th measurement element in the partitioned update Kalman filter [21]. When the state variables and the measurement variables do not belong to strict Gaussian distribution, such as in the seamless indoor/outdoor multi-source fusion positioning problem [22], the estimation error can be measured by the relative entropy. Relative entropy can also be used to calculate the number of particles in the unscented particle filter for mobile robot self-localization [23] and to calculate the sample window size in the cubature Kalman filter (CKF) [24] for attitude estimation [25]. Moreover, it has been verified that the original Kalman filter can be derived by maximizing the relative entropy [26]. Meanwhile, the robust maximum correntropy criterion has been adopted as the optimal criterion to derive the maximum correntropy Kalman filter [27,28]. However, there has been no work on the direct connections between the Rényi entropy and the Kalman filter theory until now.

In this paper, we propose a new perspective of the Kalman filter from the Rényi entropy for the first time, which bridges the gap between the Kalman filter and the Rényi entropy. We calculate the temporal derivative of the Rényi entropy for the Kalman filter mean square error matrix, which provides the optimal recursive solution mathematically and will be minimized to obtain the Kalman filter gain. Moreover, from the physical point of view, the continuous Kalman filter approaches a steady state when the temporal derivative of the Rényi entropy is equal to zero, which also means that the Rényi entropy will keep stable. A numerical experiment of falling body tracking in noisy conditions with radar using the UKF and a practical experiment of loosely-coupled integration are provided to demonstrate the effectiveness of the above conclusion.

The structure of this paper is as follows. In Section II, the definitions and properties of Shannon entropy and Rényi entropy are presented. In Section III, the Kalman filter is derived from the perspective of minimizing the temporal derivative of Rényi entropy, and the connection between the Rényi entropy and the algebraic Riccati equation is explained. In Section IV, experimental results and analysis are given by the simulation of the UKF and the real integrated navigation data. We finally conclude this paper and provide an outlook for future work in Section V.

## 2. The Connection between the Kalman Filter and the Temporal Derivative of the Rényi Entropy

### 2.1. Rényi Entropy

To calculate the Rényi entropy of the continuous probability density function (PDF), it is necessary to extend the definition of the Rényi entropy to the continuous form. The Rényi entropy of order α for a continuous random variable with a multivariate Gaussian PDF p(x) is defined [4] and calculated [9] as:

(1) $$\begin{array}{cc} H_{R}^{\alpha }\left(x\right) & =\frac{1}{1-\alpha }\ln\int_{S}p^{\alpha }\left(x\right)dx=\frac{N}{2}\ln\left(2\pi \alpha^{\frac{1}{\alpha -1}}\right)+\frac{1}{2}\ln\left(\det\Sigma \right), \end{array}$$

where α>0,α≠1, and α is a parameter providing a family of entropy functions. *N* is the dimension of the random variable *x*. S is the support. Σ is the covariance matrix of p(x).

It is straightforward to show that the temporal derivative of the Rényi entropy is given by [9]:

(2) $${\dot{H}}_{R}^{\left(\alpha \right)}\left(x\right)=\frac{1}{2}Tr\left\{\Sigma^{-1}\dot{\Sigma }\right\},$$

where $\dot{\Sigma }$ is the temporal derivative of the covariance matrix and Tr(·) is the trace operator.

It is easy to get the Shannon entropy for the multivariate Gaussian PDF by taking the limitation of Equation (1) as α approaches 1. This entropy is given as $H\left(x\right)=\frac{N}{2}\ln\left(2\pi e\right)+\frac{1}{2}\ln\left(\det\Sigma \right)$, and the temporal derivative of the Shannon entropy is given as $\dot{H}\left(x\right)=\frac{1}{2}Tr\left\{\Sigma^{-1}\dot{\Sigma }\right\}$. It is obvious the temporal of the Shannon entropy is the same as the temporal of the Rényi entropy. Therefore, we will see later that the conclusion can also be derived from the temporal derivative of the Shannon entropy. However, the Rényi entropy for the multivariate Gaussian PDF instead of the temporal derivative of the Rényi entropy will be used by adjusting the free parameter α for different uncertainty measurements in most cases, as the filtering problem has to account for the nonlinearity and the non-Gaussian noise; we adopt the Rényi entropy as the measurement for uncertainty.

### 2.2. Kalman Filter

Given the continuous-time linear system [29]:

(3) $$\dot{X}\left(t\right)=F\left(t\right)X\left(t\right)+G\left(t\right)w\left(t\right)$$

(4) Z(t)=H(t)X(t)+v(t),

where X(t) is the state vector; F(t) is the state transition matrix; G(t) is the system noise driving matrix; Z(t) is the measurement vector; H(t) is the measurement matrix; and w(t) and v(t) are independent white Gaussian noise with zero mean value; their covariance matrices are Q(t) and R(t), respectively:

(5) $$E\left[w\left(t\right)\right]=0,E\left[w\left(t\right)w^{T}\left(\tau \right)\right]=Q\left(t\right)\delta \left(t-\tau \right)$$

(6) $$E\left[v\left(t\right)\right]=0,E\left[v\left(t\right)v^{T}\left(\tau \right)\right]=R\left(t\right)\delta \left(t-\tau \right)$$

(7) $$E[w\left(t\right)v^{T}\left(\tau \right)]=0,$$

where δ(t) is the Dirac impulse function, Q(t) is a symmetric non-negative definite matrix, and R(t) is a symmetric positive matrix.

The continuous Kalman filter can be deduced by taking the limit of the discrete Kalman filter. The discrete-time state-space model is arranged as follows [29]:

(8) $$X_{k}=\Phi_{k|k-1}X_{k-1}+\Gamma_{k|k-1}W_{k-1}$$

(9) $$Z_{k}=H_{k}X_{k}+V_{k}$$

where $X_{k}$ is an n-dimensional state vector; $Z_{k}$ is an m-dimensional measurement vector; $\Phi_{k|k-1}$, $\Gamma_{k|k-1}$, and $H_{k}$ are the known system structure parameters, which are called the n×n dimensional one-step state update matrix, the n×l dimensional system noise distribution matrix, and the m×n dimensional measurement matrix, respectively; $W_{k-1}$ is the *l*-dimensional system noise vector, and $V_{k}$ is the m-dimensional measurement noise vector. Both of them are Gaussian noise vector sequences with zero mean value, and are independent of each other:

(10) $$E\left[W_{k}\right]=0,E\left[W_{k}W_{j}^{T}\right]=Q_{k}\delta_{kj}$$

(11) $$E\left[V_{k}\right]=0,E\left[V_{k}V_{j}^{T}\right]=R_{k}\delta_{kj}$$

(12) $$E[W_{k}V_{j}^{T}]=0.$$

The above equation is the basic assumption for the noise requirement in the Kalman filtering state space model, where $Q_{k}$ is a symmetric non-negative definite matrix, and $R_{k}$ is a symmetric positive definite matrix. $\delta_{kj}$ is the Kronecker δ function.

The covariance parameters $Q_{k}$ and $R_{k}$ play roles similar to those of Q and R in the continuous filter, but they do not have the same numerical values. Next, the relationship between the corresponding continuous and discrete filter parameters will be derived.

To achieve the transformation from the continuous form to the discrete form, the relations between Q and R and the corresponding $Q_{k}$ and $R_{k}$ for a small step size $T_{s}$ are needed. According to the linear system theory, the relation between *Q* and $Q_{k}$ from Equation (3) to Equation (8) is as follows:

(13) $$\Phi_{k|k-1}=\Phi \left(t_{k},t_{k-1}\right)\approx e^{\int_{t_{k-1}}^{t_{k}}F\left(\tau \right)d\tau }$$

(14) $$\Gamma_{k|k-1}W_{k-1}=\int_{t_{k-1}}^{t_{k}}\Phi \left(t_{k},\tau \right)G\left(\tau \right)w\left(t\right)d\tau .$$

Denote the discrete-time interval as $T_{s}=t_{k}-t_{k-1}$, when F(t) does not change too dramatically within the shorter integral interval $\left[t_{k-1},t_{k}\right]$. Take the Taylor expansion of $e^{F\left(t_{k-1}\right)T_{s}}$ with respect to $F\left(t_{k-1}\right)T_{s}$ and set $F\left(t_{k-1}\right)T_{s}<<I$, so the higher-order terms are negligible and the one-step transition matrix, Equation (13), can be approximated as:

(15) $$\begin{array}{cc} \; & \;\Phi_{k|k-1}\approx e^{F\left(t_{k-1}\right)T_{s}}=I+F\left(t_{k-1}\right)T_{s}+F^{2}\left(t_{k-1}\right)\frac{T_{s}^{2}}{2!}+F^{3}\left(t_{k-1}\right)\frac{T_{s}^{3}}{3!}+\cdots \approx I+F\left(t_{k-1}\right)T_{s}. \end{array}$$

Equation (14) shows that $\Gamma_{k|k-1}W_{k-1}$ is the linear transform of the Gaussian white noise w(τ); the result remains the normal distribution random vector. Therefore, the first- and second-order statistical characteristics can be used to describe and be equivalent to $\Gamma_{k|k-1}W_{k-1}$. Referring to Equation (5), the mean of $\Gamma_{k|k-1}W_{k-1}$ is given as follows:

(16) $$\begin{array}{cc} \; & \;E\left[\Gamma_{k|k-1}W_{k-1}\right]=E\left[\int_{t_{k-1}}^{t_{k}}\Phi \left(t_{k},\tau \right)G\left(\tau \right)w\left(\tau \right)d\tau \right]=\int_{t_{k-1}}^{t_{k}}\Phi \left(t_{k},\tau \right)G\left(\tau \right)E\left[w\left(\tau \right)\right]d\tau =0. \end{array}$$

For the second-order statistical characteristics, when k≠j, the time parameter between the noise $w(\tau_{k})$ and $w(\tau_{j})$ is independent, so $\Gamma_{k|k-1}W_{k-1}$ and $\Gamma_{j|j-1}W_{j-1}$ are uncorrelated:

(17) $$E\left[\left(\Gamma_{k|k-1}W_{k-1}\right){\left(\Gamma_{j|j-1}W_{j-1}\right)}^{T}\right]=0\;\left(k\neq j\right).$$

When k=j, thus

(18) $$\begin{array}{cc} E[\left(\Gamma_{k|k-1}W_{k-1}\right){\left(\Gamma_{k|k-1}W_{k-1}\right)}^{T}] & =E\left\{\left[\int_{t_{k-1}}^{t_{k}}\Phi \left(t_{k},\tau \right)G\left(\tau \right)w\left(\tau \right)d\tau \right]{\left[\int_{t_{k-1}}^{t_{k}}\Phi \left(t_{k},s\right)G\left(s\right)w\left(s\right)ds\right]}^{T}\right\}\\ \; & =E\left\{\int_{t_{k-1}}^{t_{k}}\Phi \left(t_{k},\tau \right)G\left(\tau \right)w\left(\tau \right)\int_{t_{k-1}}^{t_{k}}w^{T}\left(s\right)G^{T}\left(s\right)\Phi^{T}\left(t_{k},s\right)dsd\tau \right\}\\ \; & =\int_{t_{k-1}}^{t_{k}}\Phi \left(t_{k},\tau \right)G\left(\tau \right)\int_{t_{k-1}}^{t_{k}}E\left[w\left(\tau \right)w^{T}\left(s\right)\right]G^{T}\left(s\right)\Phi^{T}\left(t_{k},s\right)dsd\tau . \end{array}$$

Substituting Equation (5) into the above equation:

(19) $$\begin{array}{cc} E[\left(\Gamma_{k|k-1}W_{k-1}\right){\left(\Gamma_{k|k-1}W_{k-1}\right)}^{T}] & =\int_{t_{k-1}}^{t_{k}}\Phi \left(t_{k},\tau \right)G\left(\tau \right)\int_{t_{k-1}}^{t_{k}}Q\left(t\right)\delta \left(\tau -s\right)G^{T}\left(s\right)\Phi^{T}\left(t_{k},s\right)dsd\tau \\ \; & =\int_{t_{k-1}}^{t_{k}}\Phi \left(t_{k},\tau \right)G\left(\tau \right)Q\left(\tau \right)G^{T}\left(\tau \right)\Phi^{T}\left(t_{k},\tau \right)d\tau . \end{array}$$

When the noise control matrix G(τ) changes slowly during the time interval $\left[t_{k-1},t_{k}\right]$, Equation (19) becomes:

(20) $$\begin{array}{cc} \; & \;E[\left(\Gamma_{k|k-1}W_{k-1}\right){\left(\Gamma_{k|k-1}W_{k-1}\right)}^{T}]\\ \; & \approx \int_{t_{k-1}}^{t_{k}}\left[I+F\left(t_{k-1}\right)\left(t_{k}-\tau \right)\right]G\left(t_{k-1}\right)Q\left(\tau \right)G^{T}\left(t_{k-1}\right){\left[I+F\left(t_{k-1}\right)\left(t_{k}-\tau \right)\right]}^{T}d\tau \\ \; & =\left[I+F\left(t_{k-1}\right)T_{s}\right]\cdot \left[G\left(t_{k-1}\right)Q\left(t_{k-1}\right)G^{T}\left(t_{k-1}\right)T_{s}\right]\cdot {\left[I+F\left(t_{k-1}\right)T_{s}\right]}^{T}\\ \; & \;+\frac{1}{12}F\left(t_{k-1}\right)G\left(t_{k-1}\right)Q\left(t_{k-1}\right)G^{T}\left(t_{k-1}\right)F{\left(t_{k-1}\right)}^{T}T_{s}^{3}\\ \; & \approx \left\{\left[I+F\left(t_{k-1}\right)T_{s}\right]G\left(t_{k-1}\right)\right\}\cdot \left[Q\left(t_{k-1}\right)T_{s}\right]\cdot {\left\{\left[I+F\left(t_{k-1}\right)T_{s}\right]G\left(t_{k-1}\right)\right\}}^{T}. \end{array}$$

When $F\left(t_{k-1}\right)T_{s}<<I$ is satisfied, the above equation can be further approximated:

(21) $$\begin{array}{cc} \; & \;E\left[\left(\Gamma_{k|k-1}W_{k-1}\right){\left(\Gamma_{k|k-1}W_{k-1}\right)}^{T}\right]\approx G\left(t_{k-1}\right)\cdot \left[Q\left(t_{k-1}\right)T_{s}\right]\cdot G^{T}\left(t_{k-1}\right). \end{array}$$

Comparing the result with Equation (10):

(22) $$\Gamma_{k|k-1}\approx \left[I+F\left(t_{k-1}\right)T_{s}\right]G\left(t_{k-1}\right)\approx G\left(t_{k-1}\right)$$

(23) $$E\left[W_{k}W_{j}^{T}\right]=Q_{k}\delta_{kj}=\left[Q\left(t_{k}\right)T_{s}\right]\delta_{kj}.$$

Notice that [29]:

(24) $$Q_{k}=Q\left(t_{k}\right)T_{s}.$$

The derivation of the equation relating to $R_{k}$ and *R* is more subtle. In the continuous model, v(t) is white, so simple sampling of Z(t) leads to measurement noise with infinite variance. Hence, in the sampling process, we have to imagine averaging the continuous measurement over the $T_{s}$ interval to get an equivalent discrete sample. This is justified because *x* is not the Gaussian white noise and can be approximately constant within the interval.

(25) $$\begin{array}{cc} Z_{k} & =\frac{1}{T_{s}}\int_{t_{k-1}}^{t_{k}}Z\left(t\right)dt=\frac{1}{T_{s}}\int_{t_{k-1}}^{t_{k}}\left[H\left(t\right)x\left(t\right)+v\left(t\right)\right]dt=H\left(t_{k}\right)x_{k}+\frac{1}{T_{s}}\int_{t_{k-1}}^{t_{k}}v\left(t\right)dt. \end{array}$$

Then, the discrete noise matrix and the continuous noise matrix are equivalent:

(26) $$V_{k}=\frac{1}{T_{s}}\int_{t_{k-1}}^{t_{k}}v\left(t\right)dt.$$

From Equation (12), we have:

(27) $$\begin{array}{cc} E[V_{k}V_{j}^{T}] & =R_{k}\delta_{kj}=\frac{1}{T_{s}^{2}}\int_{t_{k-1}}^{t_{k}}\int_{t_{j-1}}^{t_{j}}E\left[v\left(\tau \right)v\left(s\right)\right]d\tau ds\\ \; & =\frac{1}{T_{s}^{2}}\int_{t_{k-1}}^{t_{k}}\int_{t_{j-1}}^{t_{j}}R\left(\tau \right)\delta \left(s-\tau \right)d\tau ds=\frac{1}{T_{s}^{2}}\int_{t_{k-1}}^{t_{k}}R\left(\tau \right)\delta_{kj}d\tau \approx \frac{R(t_{k})}{T_{s}}\delta_{kj}. \end{array}$$

Comparing it with Equation (6), we have [29]:

(28) $$R_{k}=\frac{R(t_{k})}{T_{s}}.$$

### 2.3. Derivation of the Kalman Filter

Assuming that the optimal state estimation at $t_{k-1}$ is ${\hat{X}}_{k-1}$, the state estimation error is ${\tilde{X}}_{k-1}$, and the state estimation covariance matrix is $\Sigma_{k-1}$:

(29) $${\tilde{X}}_{k-1}=X_{k-1}-{\hat{X}}_{k-1}$$

and

(30) $$\begin{array}{cc} \Sigma_{k-1} & =E\left[{\tilde{X}}_{k-1}{\tilde{X}}_{k-1}^{T}\right]=E\left[\left(X_{k-1}-{\hat{X}}_{k-1}\right){\left(X_{k-1}-{\hat{X}}_{k-1}\right)}^{T}\right]. \end{array}$$

If we take the expectation operator of both sides of Equation (8), we obtain the state one-step prediction and the state one-step estimation error:

(31) $$\begin{array}{cc} X_{k|k-1}^{-}=E\left[X_{k}\right] & =E\left[\Phi_{k|k-1}X_{k-1}+\Gamma_{k|k-1}W_{k-1}\right]=\Phi_{k|k-1}E\left[X_{k-1}\right]=\Phi_{k|k-1}{\hat{X}}_{k-1}, \end{array}$$

(32) $${\tilde{X}}_{k|k-1}=X_{k}-X_{k|k-1}^{-}.$$

Substituting Equations (8) and (31) into Equation (32) leads to:

(33) $$\begin{array}{cc} {\tilde{X}}_{k|k-1} & =\left(\Phi_{k|k-1}X_{k-1}+\Gamma_{k|k-1}W_{k-1}\right)-\Phi_{k|k-1}{\hat{X}}_{k-1}\\ \; & =\Phi_{k|k-1}\left(X_{k-1}-{\hat{X}}_{k-1}\right)+\Gamma_{k|k-1}W_{k-1}=\Phi_{k|k-1}{\tilde{X}}_{k-1}+\Gamma_{k|k-1}W_{k-1}. \end{array}$$

Since ${\tilde{X}}_{k-1}$ is uncorrelated with $W_{k-1}$, we therefore obtain the covariance of the state one-step estimation error ${\tilde{X}}_{k|k-1}$ as follows:

(34) $$\begin{array}{cc} \Sigma_{k|k-1} & =E\left[{\tilde{X}}_{k|k-1}{\tilde{X}}_{k|k-1}^{T}\right]=E\left[\left(\Phi_{k|k-1}{\tilde{X}}_{k-1}+\Gamma_{k|k-1}W_{k-1}\right){\left(\Phi_{k|k-1}{\tilde{X}}_{k-1}+\Gamma_{k|k-1}W_{k-1}\right)}^{T}\right]\\ \; & =\Phi_{k|k-1}E\left[{\tilde{X}}_{k-1}{\tilde{X}}_{k-1}^{T}\right]\Phi_{k|k-1}^{T}+\Gamma_{k|k-1}E\left[W_{k-1}W_{k-1}^{T}\right]\Gamma_{k|k-1}^{T}\\ \; & =\Phi_{k|k-1}\Sigma_{k-1}\Phi_{k|k-1}^{T}+\Gamma_{k|k-1}Q_{k-1}\Gamma_{k|k-1}^{T}. \end{array}$$

In a similar way, the measurement at $t_{k}$ can be predicted by the state one-step estimation prediction $X_{k|k-1}^{-}$ and system measurement Equation (9) as follows:

(35) $$Z_{k|k-1}^{-}=E\left[H_{k}X_{k|k-1}^{-}+V_{k}\right]=H_{k}X_{k|k-1}^{-}.$$

In fact, there is difference between the measurement one-step prediction $Z_{k|k-1}^{-}$ and the actual measurement $Z_{k}$. The difference is denoted as measurement one-step prediction error:

(36) $${\tilde{Z}}_{k|k-1}=Z_{k}-Z_{k|k-1}^{-}.$$

Substituting the measurement Equations (9) and (35) into Equation (36) yields:

(37) $$\begin{array}{cc} {\tilde{Z}}_{k|k-1} & =Z_{k}-H_{k}X_{k|k-1}^{-}=H_{k}X_{k}+V_{k}-H_{k}X_{k|k-1}^{-}=H_{k}{\tilde{X}}_{k|k-1}+V_{k}. \end{array}$$

In general, the measurement one-step prediction error ${\tilde{Z}}_{k|k-1}$ is called innovation in the classical Kalman filter theory, and it indicates the new information about the state estimate carried by the measurement one-step prediction error.

On the one hand, if the estimation of $X_{k}$ only includes the state one-step prediction $X_{k|k-1}^{-}$ of the system state equation, the estimation accuracy will be low, as no information of the measurement equation has been used. On the other hand, according to Equation (37), the measurement one-step prediction error calculated using the system measurement equation contains the information of the state one-step prediction of $X_{k|k-1}^{-}$. Consequently, it is natural to consider all the state information that comes from the system state equation and the measurement equation, respectively, and correct the state one-step prediction mean $X_{k|k-1}^{-}$ with the measurement one-step prediction error ${\tilde{Z}}_{k|k-1}$. Thereby, the optimal estimation of $X_{k}$ can be calculated by the combination of $X_{k|k-1}^{-}$ and ${\tilde{Z}}_{k|k-1}$ as follows:

(38) $${\hat{X}}_{k}=X_{k|k-1}^{-}+K_{k}{\tilde{Z}}_{k|k-1},$$

where $K_{k}$ is the undetermined correction factor matrix.

Substituting Equations (31) and (37) into Equation (38) obtains:

(39) $$\begin{array}{cc} {\hat{X}}_{k} & =X_{k|k-1}^{-}+K_{k}\left(Z_{k}-H_{k}X_{k|k-1}^{-}\right)=\left(I-K_{k}H_{k}\right)X_{k|k-1}^{-}+K_{k}Z_{k}\\ \; & =\left(I-K_{k}H_{k}\right)\Phi_{k|k-1}{\hat{X}}_{k-1}+K_{k}Z_{k}. \end{array}$$

From Equation (39), the current state estimation ${\hat{X}}_{k}$ is a linear combination of the last state estimation ${\hat{X}}_{k-1}$ and the current measurement $Z_{k}$, which considers the influence of the structural parameters $\Phi_{k|k-1}$ in the state equation and the structure parameters $H_{k}$ in the measurement equation with different types of construction.

The state estimation error at the current time $t_{k}$ is denoted as:

(40) $${\tilde{X}}_{k}=X_{k}-{\hat{X}}_{k},$$

where $X_{k}$ is the true values and ${\hat{X}}_{k}$ is the posterior estimation of $X_{k}$.

Substituting Equation (39) into Equation (40) obtains:

(41) $$\begin{array}{cc} {\tilde{X}}_{k} & =X_{k}-\left[X_{k|k-1}^{-}+K_{k}\left(Z_{k}-H_{k}X_{k|k-1}^{-}\right)\right]=\left(X_{k}-X_{k|k-1}^{-}\right)-K_{k}\left(H_{k}X_{k}+V_{k}-H_{k}X_{k|k-1}^{-}\right)\\ \; & ={\tilde{X}}_{k|k-1}-K_{k}\left(H_{k}{\tilde{X}}_{k|k-1}+V_{k}\right)=\left(I-K_{k}H_{k}\right){\tilde{X}}_{k|k-1}-K_{k}V_{k}. \end{array}$$

Then, the mean square error matrix of state estimation ${\hat{X}}_{k}$ is given by:

(42) $$\begin{array}{cc} \Sigma_{k} & =E\left[{\tilde{X}}_{k}{\tilde{X}}_{k}^{T}\right]=E\left\{\left[\left(I-K_{k}H_{k}\right){\tilde{X}}_{k|k-1}-K_{k}V_{k}\right]{\left[\left(I-K_{k}H_{k}\right){\tilde{X}}_{k|k-1}-K_{k}V_{k}\right]}^{T}\right\}\\ \; & =\left(I-K_{k}H_{k}\right)E\left[{\tilde{X}}_{k|k-1}{\tilde{X}}_{k|k-1}^{T}\right]{\left(I-K_{k}H_{k}\right)}^{T}+K_{k}E\left[V_{k}V_{k}^{T}\right]K_{k}^{T}\\ \; & =\left(I-K_{k}H_{k}\right)\Sigma_{k|k-1}{\left(I-K_{k}H_{k}\right)}^{T}+K_{k}R_{k}K_{k}^{T}. \end{array}$$

Substituting Equation (34) into Equation (42) obtains:

(43) $$\begin{array}{cc} \Sigma_{k} & =\left(I-K_{k}H_{k}\right)\left[\Phi_{k|k-1}\Sigma_{k-1}\Phi_{k|k-1}^{T}+\Gamma_{k|k-1}Q_{k-1}\Gamma_{k|k-1}^{T}\right]{\left(I-K_{k}H_{k}\right)}^{T}+K_{k}R_{k}K_{k}^{T}\\ \; & =\Phi_{k|k-1}\Sigma_{k-1}\Phi_{k|k-1}^{T}+K_{k}H_{k}\Phi_{k|k-1}\Sigma_{k-1}\Phi_{k|k-1}^{T}H_{k}^{T}K_{k}^{T}-\Phi_{k|k-1}\Sigma_{k-1}\Phi_{k|k-1}^{T}H_{k}^{T}K_{k}^{T}\\ \; & \;-K_{k}H_{k}\Phi_{k|k-1}\Sigma_{k-1}\Phi_{k|k-1}^{T}+\Gamma_{k|k-1}Q_{k-1}\Gamma_{k|k-1}^{T}-K_{k}H_{k}\Gamma_{k|k-1}Q_{k-1}\Gamma_{k|k-1}^{T}\\ \; & \;-\Gamma_{k|k-1}Q_{k-1}\Gamma_{k|k-1}^{T}H_{k}^{T}K_{k}^{T}+K_{k}H_{k}\Gamma_{k|k-1}Q_{k-1}\Gamma_{k|k-1}^{T}H_{k}^{T}K_{k}^{T}+K_{k}R_{k}K_{k}^{T}. \end{array}$$

We now use the approximation $\Phi_{k|k-1}\approx I+F\left(t_{k-1}\right)T_{s}$ as Equation (15). From Equation (22) with $\Gamma_{k|k-1}\approx G\left(t_{k-1}\right)$, we have:

(44) $$\begin{array}{cc} \Sigma_{k} & =\left[I+F\left(t_{k-1}\right)T_{s}\right]\Sigma_{k-1}{\left[I+F\left(t_{k-1}\right)T_{s}\right]}^{T}]+K_{k}H_{k}\left[I+F\left(t_{k-1}\right)T_{s}\right]\Sigma_{k-1}{\left[I+F\left(t_{k-1}\right)T_{s}\right]}^{T}H_{k}^{T}K_{k}^{T}\\ \; & \;-\left[I+F\left(t_{k-1}\right)T_{s}\right]\Sigma_{k-1}{\left[I+F\left(t_{k-1}\right)T_{s}\right]}^{T}H_{k}^{T}K_{k}^{T}-K_{k}H_{k}\left[I+F\left(t_{k-1}\right)T_{s}\right]\Sigma_{k-1}{\left[I+F\left(t_{k-1}\right)T_{s}\right]}^{T}\\ \; & \;+G\left(t_{k-1}\right)Q_{k-1}G^{T}\left(t_{k-1}\right)-K_{k}H_{k}G\left(t_{k-1}\right)Q_{k-1}G^{T}\left(t_{k-1}\right)-G\left(t_{k-1}\right)Q_{k-1}G^{T}\left(t_{k-1}\right)H_{k}^{T}K_{k}^{T}\\ \; & \;+K_{k}H_{k}G\left(t_{k-1}\right)Q_{k-1}G^{T}\left(t_{k-1}\right)H_{k}^{T}K_{k}^{T}+K_{k}R_{k}K_{k}^{T}. \end{array}$$

Note from Equation (24) that $Q_{k}$ is of the order of $T_{s}$ and from Equation (28) that $R_{k}=\frac{R(t_{k})}{T_{s}}$; then, Equation (44) becomes:

(45) $$\begin{array}{cc} \Sigma_{k} & =\left[I+F\left(t_{k-1}\right)T_{s}\right]\Sigma_{k-1}{\left[I+F\left(t_{k-1}\right)T_{s}\right]}^{T}]+K_{k}H_{k}\left[I+F\left(t_{k-1}\right)T_{s}\right]\Sigma_{k-1}{\left[I+F\left(t_{k-1}\right)T_{s}\right]}^{T}H_{k}^{T}K_{k}^{T}\\ \; & \;-\left[I+F\left(t_{k-1}\right)T_{s}\right]\Sigma_{k-1}{\left[I+F\left(t_{k-1}\right)T_{s}\right]}^{T}H_{k}^{T}K_{k}^{T}-K_{k}H_{k}\left[I+F\left(t_{k-1}\right)T_{s}\right]\Sigma_{k-1}{\left[I+F\left(t_{k-1}\right)T_{s}\right]}^{T}\\ \; & \;+G\left(t_{k-1}\right)Q\left(t_{k}\right)T_{s}G^{T}\left(t_{k-1}\right)-K_{k}H_{k}G\left(t_{k-1}\right)Q\left(t_{k}\right)T_{s}G^{T}\left(t_{k-1}\right)\\ \; & \;-G\left(t_{k-1}\right)Q\left(t_{k}\right)T_{s}G^{T}\left(t_{k-1}\right)H_{k}^{T}K_{k}^{T}+K_{k}H_{k}G\left(t_{k-1}\right)Q\left(t_{k}\right)T_{s}G^{T}\left(t_{k-1}\right)H_{k}^{T}K_{k}^{T}+K_{k}\frac{R(t_{k})}{T_{s}}K_{k}^{T}. \end{array}$$

### 2.4. The Temporal Derivative of the Rényi Entropy and the Kalman Filter Gain

To obtain the continuous form of covariance matrix Σ, the limit will be taken. However, the relation between the undetermined correction factor matrix $K_{k}$ and its continuous form still remains unknown. Therefore, we make the following assumption.

**Assumption** **1.** *$K_{k}$ is of the order of $T_{s}$, that is:*

(46) $$K\left(t_{k}\right)=\frac{K_{k}}{T_{s}}.$$

From the conclusion, we can also derive this assumption conversely. We next draw the conclusion as one theorem under the assumption, as follows:

**Theorem** **1.** *The discrete form of the undetermined correction factor matrix is the same as the continuous form when the temporal derivative of Rényi entropy is minimized. This can be presented in a mathematical form as follows:*

(47) $$\left\{K_{k}=\Sigma_{k}H_{k}^{T}R_{k},K=\Sigma H^{T}R^{-1}|K^{*}=\underset{K}{\arg\min}{\dot{H}}_{R}^{\left(\alpha \right)}\left(K\right)\right\}.$$

**Proof of Theorem** **1.** We substitute the expression for $K_{k}$ into Equation (45) and neglect higher-order terms in $T_{s}$; Equation (45) becomes:

(48) $$\begin{array}{cc} \Sigma_{k} & =\left[I+F\left(t_{k-1}\right)T_{s}\right]\Sigma_{k-1}{\left[I+F\left(t_{k-1}\right)T_{s}\right]}^{T}]+T_{s}K\left(t_{k}\right)H_{k}\Sigma_{k-1}\left[I+F\left(t_{k-1}\right)T_{s}\right]\Sigma_{k-1}\\ \; & \;{\left[I+F\left(t_{k-1}\right)T_{s}\right]}^{T}H_{k}^{T}T_{s}K^{T}\left(t_{k}\right)-\left[I+F\left(t_{k-1}\right)T_{s}\right]\Sigma_{k-1}{\left[I+F\left(t_{k-1}\right)T_{s}\right]}^{T}H_{k}^{T}T_{s}K^{T}\left(t_{k}\right)\\ \; & \;-T_{s}K\left(t_{k}\right)H_{k}\left[I+F\left(t_{k-1}\right)T_{s}\right]\Sigma_{k-1}{\left[I+F\left(t_{k-1}\right)T_{s}\right]}^{T}+G\left(t_{k-1}\right)Q\left(t_{k}\right)T_{s}G^{T}\left(t_{k-1}\right)\\ \; & \;-T_{s}K\left(t_{k}\right)H_{k}G\left(t_{k-1}\right)Q\left(t_{k}\right)T_{s}G^{T}\left(t_{k-1}\right)-G\left(t_{k-1}\right)Q\left(t_{k}\right)T_{s}G^{T}\left(t_{k-1}\right)H_{k}^{T}T_{s}K^{T}\left(t_{k}\right)\\ \; & \;+T_{s}K\left(t_{k}\right)H_{k}G\left(t_{k-1}\right)Q\left(t_{k}\right)T_{s}G^{T}\left(t_{k-1}\right)H_{k}^{T}T_{s}K^{T}\left(t_{k}\right)+T_{s}K\left(t_{k}\right)\frac{R_{k}}{T_{s}}T_{s}K^{T}\left(t_{k}\right)\\ \; & =\Sigma_{k-1}+T_{s}F\left(t_{k-1}\right)\Sigma_{k-1}+T_{s}\Sigma_{k-1}F^{T}\left(t_{k-1}\right)-\Sigma_{k-1}H_{k}^{T}T_{s}K{\left(t_{k}\right)}^{T}-T_{s}K\left(t_{k}\right)H_{k}\Sigma_{k-1}\\ \; & \;+G\left(t_{k-1}\right)Q\left(t_{k}\right)T_{s}G^{T}\left(t_{k-1}\right)+T_{s}K\left(t_{k}\right)\frac{R(t_{k})}{T_{s}}T_{s}K^{T}\left(t_{k}\right). \end{array}$$

Moving the first term of Equation (48) from right to left and dividing both sides by $T_{s}$ to form the finite difference expression:

(49) $$\begin{array}{cc} \frac{\Sigma_{k}-\Sigma_{k-1}}{T_{s}} & =F\left(t_{k-1}\right)\Sigma_{k-1}+\Sigma_{k-1}F^{T}\left(t_{k-1}\right)-\Sigma_{k-1}H_{k}^{T}K{\left(t_{k}\right)}^{T}-K\left(t_{k}\right)H_{k}\Sigma_{k-1}\\ \; & \;+G\left(t_{k-1}\right)Q\left(t_{k}\right)G^{T}\left(t_{k-1}\right)+K\left(t_{k}\right)R\left(t_{k}\right)K^{T}\left(t_{k}\right). \end{array}$$

Finally, passing to the limit as $T_{s}\to 0$ and dropping of the subscripts lead to the matrix differential equation:

(50) $$\begin{array}{cc} \dot{\Sigma } & =F\Sigma +\Sigma F^{T}-\Sigma H^{T}K^{T}-KH\Sigma +GQG^{T}+KRK^{T}. \end{array}$$

Σ is invertible, as it is a positive matrix. Multiplying $\Sigma^{-1}$ with Equation (50), we can consider the temporal derivative of the Rényi entropy of the mean square error matrix Σ using Equation (2):

(51) $$\begin{array}{cc} {\dot{H}}_{R}^{\left(\alpha \right)} & =\frac{1}{2}Tr\left\{\Sigma^{-1}\dot{\Sigma }\right\}\\ \; & =\frac{1}{2}Tr\left\{\Sigma^{-1}F\Sigma +F^{T}-H^{T}K^{T}-\Sigma^{-1}KH\Sigma +\Sigma^{-1}GQG^{T}+\Sigma^{-1}KRK^{T}\right\}\\ \; & =\frac{1}{2}Tr\left\{F+F^{T}-H^{T}K^{T}-KH+\Sigma^{-1}GQG^{T}+\Sigma^{-1}KRK^{T}\right\}\\ \; & =\frac{1}{2}Tr\left\{2F-2KH+\Sigma^{-1}GQG^{T}+\Sigma^{-1}KRK^{T}\right\}, \end{array}$$

where the invariance under the cyclic permutation property of the trace operator has been used to eliminate $\Sigma^{-1}$ and Σ, as well as the truth that $Tr\left(F\right)=Tr\left(F^{T}\right)$ has been used to simplify the formula.

It is obvious that Equation (51) is a quadratic function of the undetermined correction factor matrix *K*. Thereby, there must be a minimum of ${\dot{H}}_{R}^{\left(\alpha \right)}\left(x\right)$ in a probabilistic sense. Taking the derivative of both sides of Equation (51) with respect to matrix *K* obtains:

(52) $$\begin{array}{cc} \frac{\partial }{\partial K}{\dot{H}}_{R}^{\left(\alpha \right)} & =-2\frac{\partial Tr(KH)}{\partial K}+\frac{\partial Tr(\Sigma^{-1}KRK^{T})}{\partial K}\\ \; & =-2H^{T}+\frac{Tr(\Sigma^{-1}KR{\left(\partial K\right)}^{T})}{\partial K}+\frac{Tr(\Sigma^{-1}\left(\partial K\right)RK^{T})}{\partial K}\\ \; & =-2H^{T}+\Sigma^{-1}KR+{\left(RK^{T}\Sigma^{-1}\right)}^{T}. \end{array}$$

In addition, since $\Sigma^{-1}$ and $R_{k}$ are symmetric matrices, the result is:

(53) $$\frac{\partial }{\partial K}{\dot{H}}_{R}^{\left(\alpha \right)}=-2H^{T}+2\Sigma^{-1}KR.$$

$R_{k}$ is invertible, as it is a positive matrix. According to the extreme value principle of the function, when the above are equal to zero, then we have:

(54) $$K=\Sigma H^{T}R^{-1}.$$

So far, we have found the analytic solution to the undetermined correction factor matrix *K*, which is called the continuous-time Kalman filter gain in the classical Kalman filter. Then, the recursive formulations of the Kalman filter can be established through the Kalman filter gain *K*. Most importantly, this implies the connection between the temporal derivative of Rényi entropy and the classical Kalman filter: The temporal derivative of the Rényi entropy is minimized when the Kalman filter gain satisfies Equation (54).

Looking back to Assumption 1 and substituting Equation (28) into Equation (54), we obtain:

(55) $$\begin{array}{cc} K(t_{k}) & =\frac{K_{k}}{T_{s}}=K=\Sigma H^{T}R^{-1}=\Sigma_{k}H_{k}^{T}R_{k}\left(T_{s}\right)=\frac{\Sigma_{k}H_{k}^{T}R_{k}}{T_{s}}. \end{array}$$

Therefore, the discrete-time Kalman filter gain can be expressed as follows:

(56) $$K_{k}=\Sigma_{k}H_{k}^{T}R_{k}.$$

□

**Remark** **1.** *The discrete-time Kalman filter gain has the same form as the continuous-time filter gain, as shown in the Equation (54). In principle, this is consistent with our intuition and proves the correctness and rationality of Assumption A1, in turn.*

**Remark** **2.** *The Kalman filter gain is equivalent to the minimization of the temporal derivative of the Rényi entropy, although it has the same result as the original Kalman filter, which is deduced under the minimum mean square error criterion.*

Substituting Equation (54) into Equation (50), we have:

(57) $$\begin{array}{cc} \dot{\Sigma } & =F\Sigma +\Sigma F^{T}-\Sigma H^{T}K^{T}-\Sigma H^{T}R^{-1}H\Sigma +GQG^{T}+\Sigma H^{T}R^{-1}RK^{T}\\ \; & =F\Sigma +\Sigma F^{T}-\Sigma H^{T}R^{-1}H\Sigma +GQG^{T}. \end{array}$$

This is a second-order nonlinear differential equation with respect to the mean square error matrix Σ, and it is commonly called the Riccati equation. This is the same result as that of the Bucy–Kalman filter [7].

If the system equation, Equation (3), and the measurement equation, Equation (4), form a linear time-invariant system with constant noise covariance, the mean square error matrix Σ may reach a steady-state value, and $\dot{\Sigma }$ may eventually reach zero. So, we have the continuous algebraic Riccati equation as follows:

(58) $$\dot{\Sigma }=F\Sigma +\Sigma F^{T}-\Sigma H^{T}R^{-1}H\Sigma +GQG^{T}=0.$$

As we can see, the time derivative of covariance at the steady state is zero; then, the temporal derivative of the Rényi entropy should also be zero:

(59) $${\dot{H}}_{R}^{\left(\alpha \right)}=0.$$

This implies that when the system approaches a stable state, the Rényi entropy approaches a steady value so that the temporal derivative of the Rényi entropy is zero. This is reasonable when the steady system owns a constant Rényi entropy, as uncertainty is stable, which follows our intuitive understanding. Consequently, it is worth noting that whether the value of the Rényi entropy is stable or not can be a validated indicator of whether the system is approaching the steady state.

## 3. Simulations and Analysis

In this section, we give two experiments to show that when the nonlinear filter system approaches the steady state, the Rényi entropy of the system approaches stability. The first experiment is a numerical example of a falling body in noisy conditions, tracked by radar [30] using the UKF. The second experiment is a practical experiment of loosely coupled integration [29]. The simulations were carried out on MATLAB 2018a running on a computer with i5-5200U, 2.20 GHz CPU, and the graphs were plotted by MATLAB.

### 3.1. Falling Body Tracking

In the example of a falling body being tracked by radar, the body falls vertically. The radar is placed at a vertical distance *L* from the body, and the radar measures the distance *y* from the radar to the body. The state-space equation of the body is given by:

(60) $$\begin{array}{cc} {\dot{x}}_{1} & =x_{2}\\ {\dot{x}}_{2} & =d+g\\ {\dot{x}}_{3} & =0, \end{array}$$

where $x_{1}$ is the height, $x_{2}$ is the velocity, $x_{3}$ is the ballistic coefficient, g=−9.81 m/s${}^{2}$ is the gravity acceleration, and *d* is the air drag, which could be approximated as:

(61) $$\begin{array}{c} d=\frac{\rho x_{2}^{2}}{2x_{3}}=\rho_{0}\exp\left(-\frac{x_{1}}{k}\right)\frac{x_{2}^{2}}{2x_{3}}, \end{array}$$

where ρ is the air density with an initial value of $\rho_{0}=1.225$; $\rho_{0}=1.225$ and k=6705.6 are constants.

The measurement equation is:

(62) $$y=\sqrt{L^{2}+x_{1}^{2}}.$$

It is worth noting that the drag and the square root cause severely nonlinearity in the state-space function and measurement function, respectively.

The discrete-time nonlinear system can be given by the Euler discretization method. Combining the additive process with Gaussian white noises for measurement, we can obtain:

(63) $$\begin{array}{cc} x_{1}\left(n+1\right) & =x_{1}\left(n\right)+x_{2}\left(n\right)\cdot T+w_{1}\left(n\right)\\ x_{2}\left(n+1\right) & =x_{2}\left(n\right)+\left(d+g\right)\cdot T+w_{2}\left(n\right)\\ x_{3}\left(n+1\right) & =x_{3}\left(n\right)+w_{3}\left(n\right) \end{array}$$

(64) $$y\left(n\right)=\sqrt{L^{2}+x_{1}^{2}\left(n\right)}+v\left(n\right).$$

**Figure 1:**
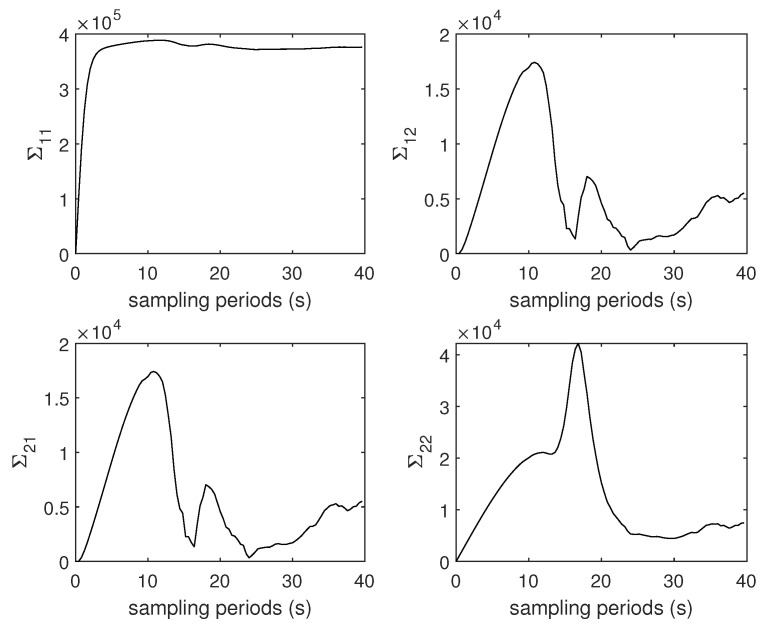
Evolution of matrix Σ.

**Figure 2:**
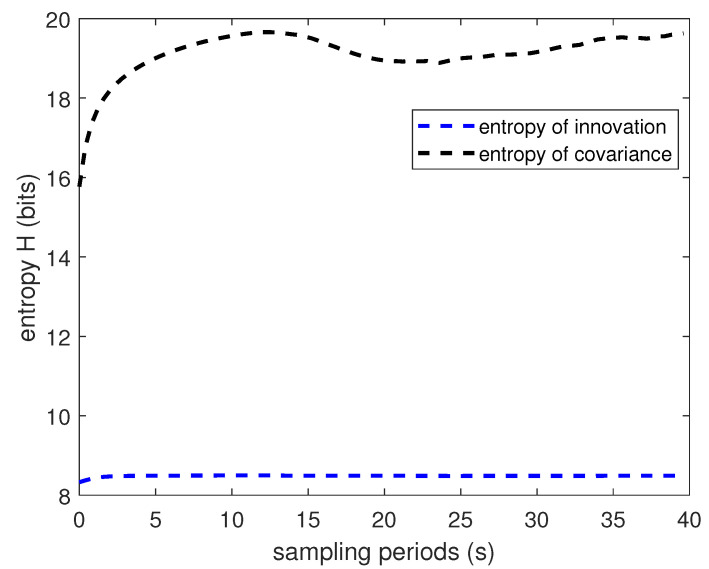
Simulation results for the entropy.

**Figure 3:**
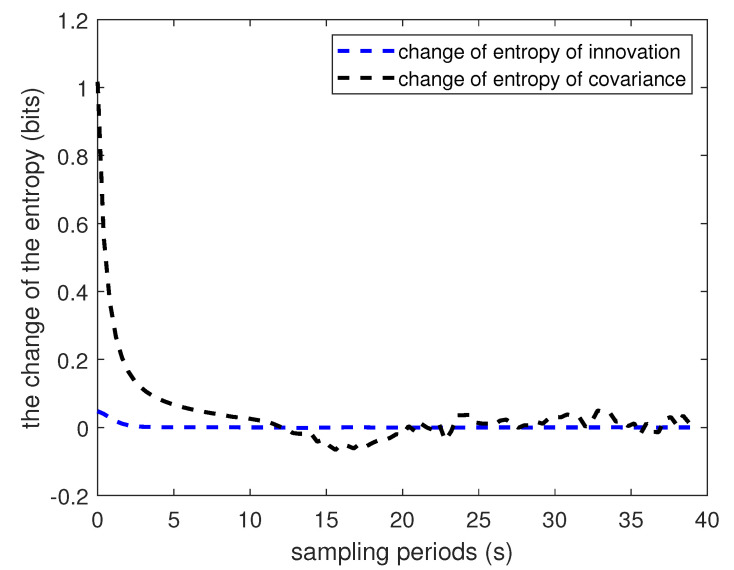
Simulation results for the change of entropy.

In the UKF numerical experiment, we set the sampling period to T=0.4 s, the horizontal distance to L=100 m, the maximum number of samples to N=100, the process noise to $S_{w}=diag\left(10^{5},10^{3},10^{2}\right)$, the measurement noise to $S_{v}=10^{6}$, and the initial state to $x=[10^{5};-5000;400]$. The results are shown as follows:

Figure 1 shows the evolution of covariance matrix Σ. Figure 2 and Figure 3 show the Rényi entropy of covariance matrix Σ and its change in adjacent time, respectively. Notice that the uncertainty increases near the middle of the plots, which is coincident with the drag peak. However, the Rényi entropy fluctuates around 15; even the fourth element of Σ changes dramatically. Of course, the entropy changes are closely accompanied by the drag peak, which means the change of the entropy of covariance reflects the evolution of matrix Σ. Consequently, the Rényi entropy can be viewed as the indicator of whether the system is approaching the steady state or not.

### 3.2. Practical Integrated Navigation

In the loosely integrated navigation system, the system state parameter *x* is composed of inertial navigation system (INS) error states in the North–East–Down (NED) local-level navigation frame, and can be expressed as follows:

(65) $$x={\left[{\left(\delta r^{n}\right)}^{T}\;{\left(\delta v^{n}\right)}^{T}\;{\left(\psi \right)}^{T}\;{\left(b_{g}\right)}^{T}\;{\left(b_{a}\right)}^{T}\right]}^{T},$$

where $\delta r^{n}$, $\delta v^{n}$, and ψ represent the position error, the velocity error, and the attitude error, respectively; $b_{g}$ and $b_{a}$ are modeled as first-order Gauss–Markov processes, representing the gyroscope bias and the accelerometer bias, respectively.

The discrete-time state update equation is used to update state parameters as follows:

(66) $$x_{k}=\Phi_{k|k-1}x_{k-1}+G_{k|k-1}w_{k-1},$$

where $G_{k|k-1}$ is the system noise matrix, $w_{k-1}$ is the system noise, and $\Phi_{k|k-1}$ is the state transition matrix from $t_{k-1}$ to $t_{k}$; this is determined by the dynamic model of the state parameter.

In the loosely coupled integration, the measurement equation can be simply expressed as:

(67) $$\delta z=H_{k}x_{k}+v_{k},$$

where $v_{k}$ is the measurement noise, $H_{k}$ is the measurement matrix, and $z_{k}$ is the measurement vector calculated by subtracting the global navigation satellite system (GNSS) observation with the inertial navigation system (INS) mechanism.

The experiments reported in this section were carried out by processing the data from an unmanned ground vehicle test. The gyroscope random walk was set to 0.03 deg/$\sqrt{h}$ and the velocity random walk was set to 0.05 m/s/$\sqrt{h}$. The sampling rates of the inertial measurement unit (IMU) and the GNSS are 200 Hz and 1 Hz, respectively. The test lasts 48 min.

**Figure 4:**
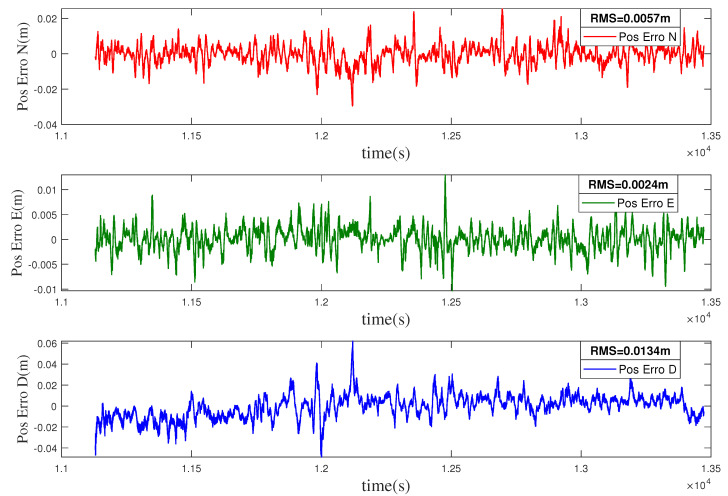
Position error of the loosely coupled integration.

**Figure 5:**
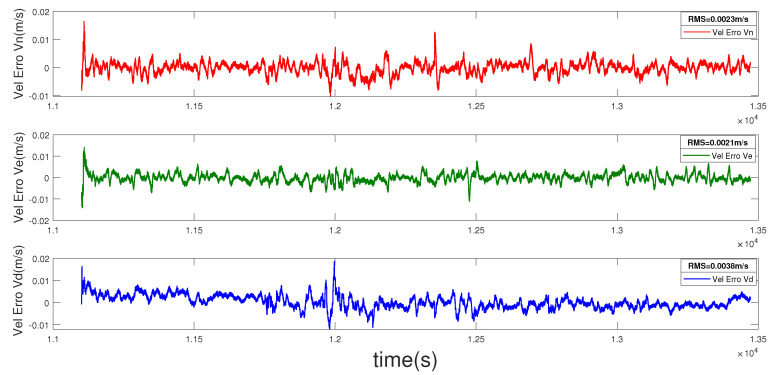
Velocity error of the loosely coupled integration.

**Figure 6:**
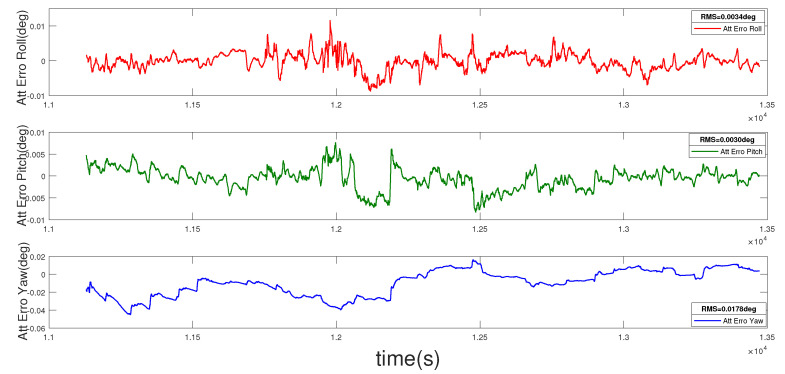
Attitude error of the loosely coupled integration.

The position error curve, velocity error curve, and attitude error curve of the loosely coupled integration are shown in Figure 4, Figure 5 and Figure 6. The root mean squares (RMSs) of the position errors in the north, east, and earth directions are 0.0057 m, 0.0024 m, and 0.0134 m, respectively. The RMS of the velocity errors in the north, east, and earth directions are 0.0023 m/s, 0.0021 m/s, and 0.0038 m/s, respectively. The RMSs of the attitude errors in the roll, pitch, and yaw directions are 0.0034 deg, 0.0030 deg, and 0.0178 deg, respectively.

**Figure 7:**
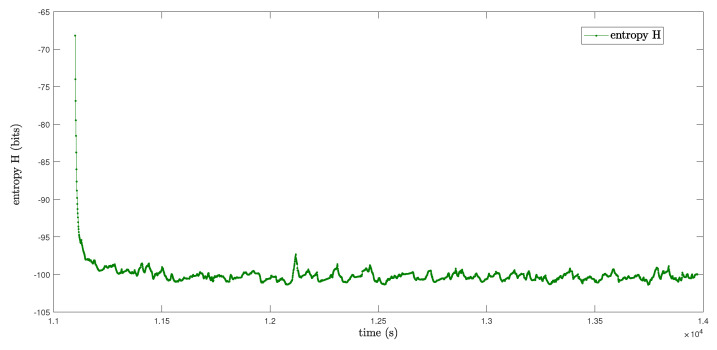
Rényi entropy of the covariance Σ.

The Rényi entropy of the covariance *P* is shown in Figure 7. As we can see, the Rényi entropy fluctuates around −100 once the filter converges, which is consistent with the conclusion from the entropy perspective.

## 4. Conclusions and Final Remarks

We have considered the original Kalman filter by taking the minimization of the temporal derivative of the Rényi entropy. In particular, we show that the temporal derivative of Rényi entropy is equal to zero when the Kalman filter system approaches the steady state, which means that the Rényi entropy approaches a stable value. Finally, simulation experiments and practical experiments show the Rényi entropy truly stays stable when the system becomes steady.

Future work includes calculating the Rényi entropy of the innovation term when the measurements and the noise are non-Gaussian [14] in order to evaluate the effectiveness of measurements and adjust the noise covariance matrix. Meanwhile, we can also calculate the Rényi entropy of the nonlinear dynamical equation to measure the nonlinearity in the propagation step.
